# Developing prehospital clinical practice guidelines for resource limited settings: why re-invent the wheel?

**DOI:** 10.1186/s13104-018-3210-3

**Published:** 2018-02-05

**Authors:** Michael McCaul, Ben de Waal, Peter Hodkinson, Jennifer L. Pigoga, Taryn Young, Lee A. Wallis

**Affiliations:** 1 Centre for Evidence-based Health Care, Division of Epidemiology and Biostatistics, Stellenbosch University, Cape Town, South Africa; 2 Department of Emergency Medical Sciences, Cape Peninsula University of Technology, Cape Town, South Africa; 3 Division of Emergency Medicine, University of Cape Town, Cape Town, South Africa; 4 Cochrane South Africa, South African Medical Research Council, Cape Town, South Africa; 5 Division of Emergency Medicine, Stellenbosch University, Cape Town, South Africa

**Keywords:** Prehospital, Emergency medicine, Emergency care, Clinical practice guidelines, Guidelines, Guideline development, Adaptation

## Abstract

**Objectives:**

Methods on developing new (de novo) clinical practice guidelines (CPGs) have received substantial attention. However, the volume of literature is not matched by research into alternative methods of CPG development using existing CPG documents—a specific issue for guideline development groups in low- and middle-income countries. We report on how we developed a context specific prehospital CPG using an alternative guideline development method. Difficulties experienced and lessons learnt in applying existing global guidelines’ recommendations to a national context are highlighted.

**Results:**

The project produced the first emergency care CPG for prehospital providers in Africa. It included > 270 CPGs and produced over 1000 recommendations for prehospital emergency care. We encountered various difficulties, including (1) applicability issues: few pre-hospital CPGs applicable to Africa, (2) evidence synthesis: heterogeneous levels of evidence classifications and (3) guideline quality. Learning points included (1) focusing on key CPGs and evidence mapping, (2) searching other resources for CPGs, (3) broad representation on CPG advisory boards and (4) transparency and knowledge translation. Re-inventing the wheel to produce CPGs is not always feasible. We hope this paper will encourage further projects to use existing CPGs in developing guidance to improve patient care in resource-limited settings.

**Electronic supplementary material:**

The online version of this article (10.1186/s13104-018-3210-3) contains supplementary material, which is available to authorized users.

## Introduction

Clinical practice guidelines (CPGs) form the cornerstone of providing synthesised systematic evidence-based guidance to patients, healthcare practitioners and managers. Methods on developing new (de novo) CPGs have, of late, received substantial attention [1]. However, the volume of literature is not matched by research into alternative methods of CPG development using existing clinical practice guidance documents [2], a specific issue for guideline development groups in low-and middle-income countries (LMICs). De novo CPG development could be out of reach for many, as it is a time-consuming and expensive process requiring multifaceted teams of methodologists and experts who systematically review and synthesise primary evidence, to ultimately produce locally appropriate recommendations. Furthermore, some argue that the higher burden of disease in LMICs makes the focus on evidence-based guidelines even more urgent, to minimise wastage and ensure the best patient care for optimal cost [2, 3].

Consequently, alternative approaches to de novo CPG development have been proposed, using existing high-quality clinical guidelines to make recommendations relevant to local contexts through a process of adopting, adapting or contextualising [2, 4]. These approaches are attractive where resources are limited, especially when high-quality guidance already exists (mostly from high-income countries) [5, 6]. However, limited examples exist in the literature to showcase the pragmatic application of such alternative approaches in settings with time and budget constraints.

South African prehospital emergency care providers are currently practicing based on protocols that are more than a decade old [7]. These protocols focus mainly on resuscitation and pharmacopeia, providing little-to-no information on background evidence, contextual application or care of patients. The South African emergency care profession has seen rapid growth, but the profession faces a particular challenge with developing guidelines as there is a myriad of pre-hospital qualifications and an inequitable workforce distribution across the country. Consequently, there has been a recent drive to develop evidence-based CPGs to replace the existing protocols with reliable and robust guidelines. This is of particular value and importance for guideline development methods in Africa, especially since prehospital care protocols to-date still use GOBSAT methods (good-old-boys-sitting around-a-table) [7] with a lack of due processes and little reliance on evidence. Developing such evidence-based guidelines would be a major step forward in adopting better and more structured practices for the guideline development footprint in sub-Saharan Africa. In this paper, we report on how we developed context specific prehospital CPGs, along with the difficulties experienced and lessons learnt in applying existing global guidelines’ recommendations to a national context.

## Main text

### About this project and process highlights

The primary focus of the project was to create a contextually appropriate evidence based CPG for prehospital emergency care providers and managers. The guideline needed to be patient-centred, realistic and enhance the continuation of care through the emergency system from prehospital to patient discharge. The detailed aims and scope of the project have been reported elsewhere [8].

Developing a comprehensive prehospital CPG is a daunting project that would take years to complete de novo. Due to limitations in time, funding and the sheer scope of the project, this was not an option. We thus adopted a novel approach allowing us to work within our resource constraints [2]. This alternative approach started via engagement with an advisory board of key stakeholders, including methodologists, prehospital providers and various medical specialists. After clarifying the clinical questions, the core guideline team (an independent working group supported by the advisory board) identified and appraised existing CPGs (as there exists internationally a wide range of high-quality international CPGs covering most of the key topics required) and then used these to develop contextually appropriate evidence-based CPGs.

Comprehensive searching for CPGs was performed and followed systematic review methods, including comprehensive searching of the literature (Additional file 1), critical appraisal and synthesis. Potential included guidelines where full text was obtained were critically appraised using the AGREE II tool [9]. The AGREE II scores were used to assess and prioritise which guidelines to include, particularly if there were two or more competing guidelines on similar topics. Within priority areas, different guideline recommendations often overlapped; in this case the most current and unambiguous recommendation was accepted. High-quality, relevant and up-to-date guidelines were prioritised through consensus by the core guideline panel. In some cases, guidelines were excluded due to extremely poor AGREE II scores, even when there was only a single guideline on the topic.

Dizon et al. describe the process of adopting, adapting or contextualising existing CPGs for local use and have set the foundation on which this CPG was formulated [2] (Additional file 2). We ‘adapted’ this method into a simpler method for formulating recommendations based on existing evidence. Any decision to adopt, adapt or contextualise was made by the core guideline panel and reviewed by the advisory board. Where applicable, ‘practice points’ were also added; these included more specific guidance to clinicians regarding how to perform a particular intervention, or provided further clarity for use at the bedside (e.g. how to prepare and administer a drug related to a particular recommendation).

**Table:** 

Process	Explanation
Adopting	Recommendations were adopted when they could be applied directly, without any changes, to the South African context. Adopting meant a commitment to implement its recommendations as proposed, without any subtle changes or caveats
Adapting	Recommendations were adapted if they required changes, updated evidence (preferably from a systematic review) or adding implementation caveats that changed the meaning of the original recommendation. Adapted recommendations are considered new recommendations and no longer have an attached level of evidence or strength of recommendations
Contextualising	Contextualising a recommendation meant not making any changes, but incorporating local context conditions integral for implementing the recommendation [2]. Contextual points included commentary around locally-appropriate alternative methods of intervention delivery, system issues that would need to be addressed, or simply caveats to the recommendation within the current emergency care system

A brief example of adopting, adapting and contextualising guidelines is presented in Additional file 3. Additional tables and figures are available as on-line supplements to this paper, and provide useful insights into processes for other countries and guideline teams.

Overall, the steps and processes are predominantly the same as for de novo guideline development. However, key differences included identifying and synthesising high-quality CPGs for emergency care, instead of primary level evidence such as randomised controlled trials or observational studies. The main differences between de novo development and this guideline approach is highlighted in Additional file 4 [1].

### Our experience

The project produced the first emergency care CPG for prehospital providers in Africa. It included more than 270 CPGs and produced over 1000 recommendations for prehospital emergency care (Additional file 5). It represents a transition from opinion-based and skills-driven practice to evidence-informed clinical practice. The guideline is currently in the implementation and dissemination phase, with national health regulatory bodies in the process of incorporating public and industry feedback on the guidelines.

We encountered various difficulties in guideline development within emergency care. We have summarised the three key factors that generated debate and uncertainty throughout the process.

#### Applicability: few pre-hospital CPGs applicable to Africa

Fewer than 1% of the 276 included CPGs originated from and were directly applicable to LMICs, as the vast majority of the data came from the United States, Europe and Australia. We found no CPGs that provided targeted recommendations for prehospital emergency care in LMIC settings. This provided a particular challenge to the process of adopting, adapting or contextualising, as in most cases the generalisability and applicability of recommendations to the local setting was unclear.

#### Evidence synthesis: heterogeneous levels of evidence classifications

Reporting adopted or contextualised guideline recommendations’ level of evidence and strength of recommendations was difficult. Different CPGs used different classification systems (e.g. GRADE or NHMRC) and as such, we found significant heterogeneity between recommendation reporting systems. This made reporting recommendations difficult, as each has a different and often indistinguishable classification system. In response to this, we opted to report the original plain language meaning for each classification. For example, level I evidence was simply reported as ‘evidence obtained from a systematic review of all relevant randomised controlled trials’, taken verbatim from the original classification description. Guideline developers should use a single, robust and clear recommendations classification system, such as GRADE.

#### Guideline quality: all are not equal

The quality of included CPGs varied significantly, with many scoring so poorly on AGREE-II that they were excluded. We screened more than 1000 ‘guidelines’, but the majority were excluded simply due to the absence of any reported methods or not using a systematic process of synthesising evidence. There was significant variation between guidelines developed by professional societies compared to collaborative groups. Guidelines from larger organisations, such as Guidelines International Network (G-I-N), National Institute for Health and Care Excellence (NICE) and the National Guidelines Clearinghouse (NGC) were often the highest quality.

### Learning points

#### Focus on key CPGs and evidence mapping

Guidelines teams should focus on selecting only a couple of key high-quality, relevant, up-to-date CPGs for adoption or adaptation to save considerable time and effort when extracting relevant recommendations. This should only be done within the background of having clearly clarified the guideline scope and prioritisation of clinical questions. Despite doing this, guidelines teams could still end up with large numbers of guidelines that have relevant recommendations linked to multiple clinical questions. To mitigate this confusion, guideline teams should map and match clinical guidelines to the a priori defined clinical questions and focus areas. This could take the form of a database or an electronic mind map, which assist with cross-referencing, identifying evidence gaps, and grouping recommendations for the various guidelines within clinical questions and overarching topics.

#### Searching other resources for CPGs

PubMed, EMBASE and equivalent medical literature databases are traditionally the first port of call for finding evidence. However, we found that these traditional databases alone yielded very poor results. Out of the 276 CPGs included, only three originated from PubMed. The majority were identified in guideline clearing houses (e.g. NGC), databases (e.g. G-I-N or NICE), or Google. Guidelines teams should thus use alternative sources to traditional electronic databases when searching for CPGs.

#### Broad representation on CPG advisory board

To promote wide input and buy-in from key role players, it was important that representatives from a range of fields within and relating to emergency care were involved [10]. These included training institutions, emergency physicians, and medical specialists involved in receiving and care of patients managed by emergency care providers. The advisory panel was involved at key points in the process. They reviewed each CPG relevant to their practice and their comments were incorporated into the final CPG to make it context-specific and ensure that it was in line with existing national guidelines and processes as best as possible.

#### Transparency and knowledge translation

Transparency in guideline development is of utmost importance, especially in complex processes and decision-making events such as incorporating advisory board input, adapting methods to respond to the political environment and engaging project sponsors. Keeping clear records of such events, decisions and processes is essential in producing a robust and trustworthy CPG. Regular feedback around processes and interim progress reports to sponsors provide mechanisms to ensure a complete product.

Although CPGs act as a vehicle for change and knowledge translation, focusing on clear dissemination and implementation strategies, including end-user content, is paramount to enabling successful uptake of guidelines. Regrettably, these elements were outside the scope of this project, as time and budget were limited; however, guideline teams should strongly consider incorporating guideline implementation and dissemination strategies as part of the initial conversation on scope and timeframe.

Looking to the future, this project seeks to further validate and strengthen alternative guideline development methods for resource-limited settings, and to conduct further research to support guideline implementers in formulating a national guideline implementation and dissemination strategy by investigating local barriers and solutions among paramedics to promote guideline uptake together with decision makers. Re-inventing the wheel to produce CPGs is not always feasible. We hope this paper will encourage further projects to use existing CPGs in developing guidance to improve patient care in resource-limited settings.

## Limitations

The African Federation for Emergency Medicine (AFEM) project only included clinical practice guidelines to adapt, adopt or contextualise that were of high-quality, up-to-date and that fit the strict Institute of Medicine definition of clinical practice guidelines. Other guidance documents such as protocols, end-user guides or patient pathways were excluded.

## Additional files

**Additional file 1.** Search strategy and example search string.

**Additional file 2.** Suitcase analogy for adopting, adapting and contextualising guidelines.

**Additional file 3.** Example recommendations: adopting, adapting and contextualising.

**Additional file 4.** De novo vs African Federation for Emergency Medicine (AFEM) alternative guideline development approach.

**Additional file 5.** Guideline inclusion flow diagram.

## Abbreviations

AFEM: The African Federation for Emergency Medicine
CPG: Clinical Practice Guideline
EMS: Emergency Medical Services
GRADE: Grades of Recommendation Assessment, Development and Evaluation
G-I-N: Guidelines International Network
HPCSA: Health Professions Council of South Africa
LMIC: Low-and middle-income countries
NGC: National Guidelines Clearinghouse
NHMRC: National Health and Medical Research Council
NICE: National Institute for Health and Care Excellence
PBEC: Professional Board of Emergency Care
SAGE: South African Guidelines Excellence Project
