# Supplementary material for: Developing prehospital clinical practice guidelines for resource limited settings: why re-invent the wheel?
Source: BMC Res Notes. 2018 Feb 5;11:97. doi: 10.1186/s13104-018-3210-3 (PMC5800053; doi:10.1186/s13104-018-3210-3)
Supplement: Supplementary file 1 — Additional file 1. Search strategy and example search string. [file 13104_2018_3210_MOESM1_ESM.docx]

S1 Search strategy and example search string

Searching was performed by members of the AFEM core guideline panel with support from the Stellenbosch University Faculty of Medicine and Health Sciences Library staff. Guidelines were searched using two phases 1) searching for prehospital-specific guidelines and 2) searching for condition-specific guidelines (e.g. stoke or trauma). Searches was conducted in Google (06 October 2015), MEDLINE (using Pubmed and MeSH terms, 27 October 2015) and the following databases using iterative pragmatic search strings:

- National Guideline Clearinghouse (06 and 08 October 2015)
- Guideline Central (08 October 2015)
- Guidelines-International-Network (07 October 2015)
- National Institute for Health and Care Excellence (08 October 2015 and 20 February 2016)
- TRIP database (08 October 2015)
- New Zealand Guidelines Group (07 October 2015)
- Australian Government National Health and Medical Research Council (07 October 2015)
- Canadian Medical Association CPG database (07 October 2015)

Websites included:

International liaison Committee on Resuscitation, American Health Association, European Resuscitation Council, Japanese Resuscitation Council, Emergency Medicine Society of South Africa, African Federation for Emergency Medicine, Emergency Nurses Society of South Africa, Royal College of Obstetricians and Gynaecologists, World Health Organisation and the Critical Care Society of South Africa.

Since guideline specific websites or professional society websites do not require a guideline search string, searching for general prehospital guidelines included search strings such as ‘prehospital’, ‘ambulance’, emergency care’, ‘out-of-hospital’, ‘emergency services’ and ‘paramedic’. Where applicable and possible, search filters were used along with relevant MeSH terms if the database or website allowed this.

Example search strings:

PUBMED (general)

((((((("Emergency Medical Services"[Mesh] OR "prehospital" OR "pre-hospital" OR "out-of-hospital" OR "out of hospital" OR "in-the-field" OR "in the field" OR "ambulance" OR "emergency ambulance" OR "emergency medical services" OR "ambulance service*" OR "paramedic" OR "Paramedic*"))) AND (("guideline*" OR "clinical practice guide*" OR "clinical practice guideline*" OR "practice guideline*" OR "practice guide* OR "patient management guide" OR "patient care guide""))))))

PUBMED (condition-specific)

(((((((("Acute Coronary Syndrome"[Mesh] OR "acute coronary syndrome" OR "ACS" OR "Myocardial Infarction"[Mesh] OR "STEMI" OR "NSTEMI" OR "ST elevation myocardial infarction" OR "Non ST elevation myocardial infarction")))))) AND ((((((("prehospital" OR "pre-hospital" OR "out-of-hospital" OR "out of hospital" OR "in-the-field" OR "in the field" OR "ambulance" OR "emergency ambulance" OR "emergency medical services" OR "ambulance service*" OR "paramedic" OR "Paramedic*"))) AND (("guideline*" OR "clinical practice guide*" OR "clinical practice guideline*"

OR "practice guideline*" OR "practice guide*")))))))
