# Supplementary figures and images for: Developing prehospital clinical practice guidelines for resource limited settings: why re-invent the wheel?
Source: BMC Res Notes. 2018 Feb 5;11:97. doi: 10.1186/s13104-018-3210-3 (PMC5800053; doi:10.1186/s13104-018-3210-3)

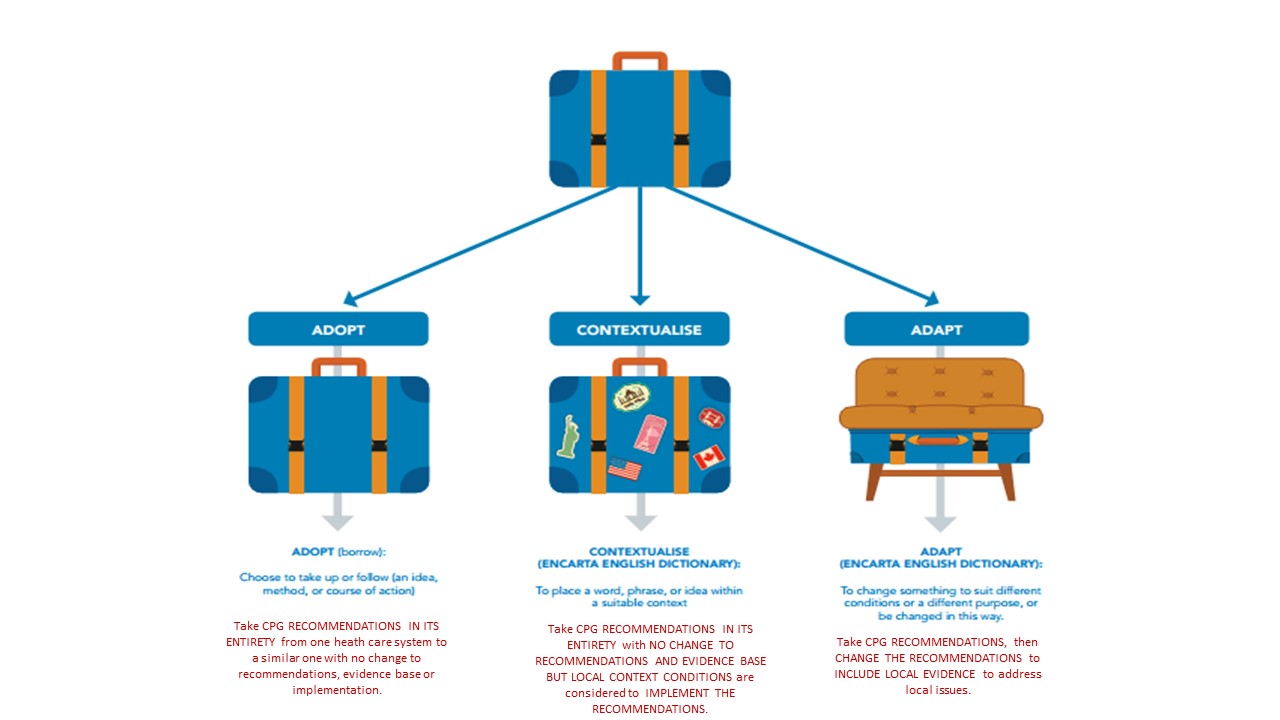

Supplement: Supplementary file 2 — Additional file 2. Suitcase analogy for adopting, adapting and contextualising guidelines. [file 13104_2018_3210_MOESM2_ESM.jpg]

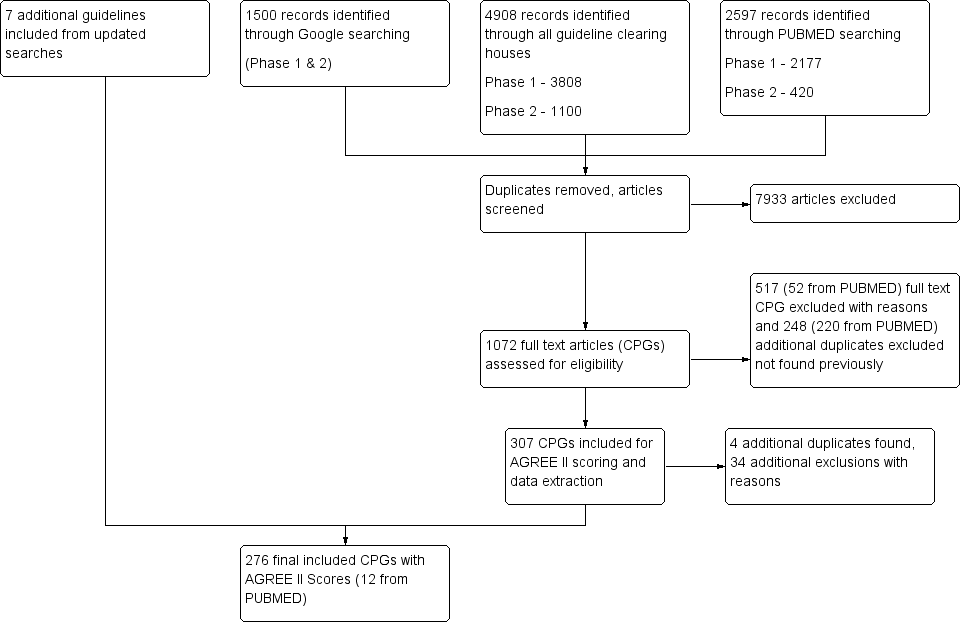

Supplement: Supplementary file 5 — Additional file 5. Guideline inclusion flow diagram. [file 13104_2018_3210_MOESM5_ESM.tif]
