# Supplementary material for: Developing prehospital clinical practice guidelines for resource limited settings: why re-invent the wheel?
Source: BMC Res Notes. 2018 Feb 5;11:97. doi: 10.1186/s13104-018-3210-3 (PMC5800053; doi:10.1186/s13104-018-3210-3)
Supplement: Supplementary file 3 — Additional file 3. Example recommendations: adopting, adapting and contextualising. [file 13104_2018_3210_MOESM3_ESM.docx]

S3 Examples of adapted and contextualised recommendations

| **Original Evidence Statement** | **Adapted recommendations** |
| --- | --- |
| Effective immobilization of fractures to minimize morbidity should be carried out and a written record of the treatment passed to the hospital | Gross alignment and effective immobilization of all fractures (Thomas splint for femur fractures and pneumatic and wire splints with addition of slings for upper limb fractures) should be carried out and a written record of the treatment passed to the hospital. To minimize morbidity neurovascular status should be checked before and after, if it should worsen in the new immobilised position the limb should be returned to the position of best distal neurovascular status. |
| Prehospital use of aspirin is recommended prior to PPCI | Prehospital use of aspirin is highly recommended prior to PPCI and mandatory for pre-hospital fibrinolysis |
| **Original Evidence Statement** | **Contextual points** |
| We recommend prehospital 12-lead ECG acquisition with hospital notification for adult patients with suspected STEMI | Providers who use 3 lead ECG must understand it’s limited diagnostic capacity in patients with STEMI. |
| PPCI: The routine transfer to facilities with 24/7 PPCI is mandatory | Where PPCI is available, otherwise transfer to most appropriate facility for thrombolysis or provide prehospital thrombolysis if indicated and available. |
